# *Serratia marcescens* colonization in preterm neonates during their neonatal intensive care unit stay

**DOI:** 10.1186/s13756-019-0584-5

**Published:** 2019-08-09

**Authors:** Laura Moles, Marta Gómez, Elena Moroder, Esther Jiménez, Diana Escuder, Gerardo Bustos, Ana Melgar, Jeniffer Villa, Rosa del Campo, Fernando Chaves, Juan M. Rodríguez

**Affiliations:** 1 Department of Nutrition and Food Science, Complutense University of Madrid, Avda. Puerta de Hierro, s/n, 28040 Madrid, Spain; 2 Servicio de Pediatría Hospital Francesc de Borja, Gandía, Valencia, Spain; 3 Servicio de Neonatología, Hospital Universitario 12 de Octubre, Madrid, Spain; 4 Red de Salud Materno-Infantil y del Desarrollo (SAMID), Instituto Carlos III, Madrid, Spain; 5 Servicio de Microbiología, Hospital Universitario 12 de Octubre, Madrid, Spain; 6 Servicio de Microbiología y Parasitología, Hospital Universitario Ramón y Cajal and Instituto Ramón y Cajal de Investigaciones Sanitarias, Madrid, Spain

**Keywords:** Prematurity - antiobiotic resistance - gut colonization - sepsis - enteral feeding tubes

## Abstract

**Background:**

Nosocomial sepsis is the main problem that preterms have to face during their stay at neonatal intensive care units (NICU). *Serratia marcescens* is an emerging cause of preterm sepsis but its epidemiology is still largely unknown. Consequently, the aims of this study were to know the rate of preterms colonized by *S. marcescens* during their stay at the NICU and the characteristics and evolution of the *S. marcescens* population, including the susceptibility to clinically relevant antibiotics.

**Methods:**

Twenty-six preterm infants born with a gestational age ≤ 32 weeks and/or weigh ≤1500 g were included in the study. Samples of meconium and feces (*n* = 92) were collected during their first month of life of the infants, together with feeding samples after their pass through enteral feeding tubes (*n* = 37). Samples were inoculated on MacConkey agar plates. The isolates identified as *S. marcescens* were genotyped using RAPD and PFGE; and antibiotics susceptibility was performed in a Vitek 2 system.

**Results:**

A total of 179 *S. marcescens* isolates were obtained from the samples. PFGE profiling and cluster analysis allowed the classification of the isolates into 7 different *S. marcescens* clones. PFGE patterns 1 and 3 were the dominant strains in the fecal samples colonizing 31 and 35% of the infants, respectively. Those isolates causing bacteremia in two infants clustered in PFGE pattern 3.

**Conclusion:**

*S. marcescens* is a bacterial species closely associated to the NICU environment. It can be frequently isolated from preterm’s feces although only some genetic lineages seem to be associated to sepsis. Enteral feeding tubes act as important reservoirs to keep the *S. marcescens* population in the NICU.

**Trial registration:**

The local ethic committee approved this trial with the reference 09/157.

## Background

Premature infants are a particularly sensitive human subpopulation group. Their immature organs, long hospitalization and altered gut microbiota, result in developmental problems and in an increased risk of diseases that may lead to life-threatening conditions [1]. Their fecal microbiota is characterized by lower diversity, higher representation of potential pathogens, and reduced, delayed and rather fluctuating colonization by strict anaerobic symbiotic bacteria [2, 3]. Recent data suggested that preterm’s microbiota alterations may precede the development of diseases as sepsis or necrotizing enterocolitis (NEC) [4, 5]. Preterms are usually exposed to bacteria well adapted to the hospital environment and that are recognized as high-risk clones [6]. Furthermore, preterms are usually treated with antibiotics, which also select the multidrug-resistant bacteria colonizing their guts.

The microbiota of preterm neonates was previously assessed by using both culture-dependent and -independent techniques [7]. Dominant species were characterized and a high proportion of antibiotic-resistant high-risk clones was detected during the NICU admittance [6]. However, *S. marcescens* isolates were excluded from the analysis because of the lack of an MLST scheme, despite being the second species (after *S. epidermidis*) on the basis of the percentage of positive infants and the third species (after *E. faecalis* and *S. epidermidis*) on the basis of the total number of isolates obtained from the samples.

In this context, the objective of this study was to know the rate of preterm neonates colonized by *S. marcescens* during their stay at the NICU in the absence of any outbreak, and to characterize the *S. marcescens* population and their evolution in the NICU.

## Methods

### Subjects and sampling

Twenty-six preterm infants born between October 2009 and June 2010 in the maternity of the Hospital Universitario 12 de Octubre (Madrid, Spain) were recruited for the study. The NICU of the Hospital is a 41-bed unit distributed in four rooms. The first room includes 19 intensive care beds, while the other 22 beds are divided up in 3 intermediate separate intensive care rooms. The mean number of births in the last 5 years in the institution was 4443 and 10.7% of them were premature. The occupation of the NICU was high, reaching a mean value of 93%. The rate of very low birth weight (VLBW) and extremely low birth weight (ELBW) infants in the same period was of 29 and 10%, respectively, while their mean survival rates were 90 and 76%, respectively. To be eligible for the study infants must to be born at a gestational age ≤ 32 weeks and/or weigh ≤1500 g. Neonates with any malformations, metabolic diseases or severe conditions were excluded. The study was approved by the local ethical committee (reference 09/157) and written informed parental consent was obtained for each infant before his/her inclusion.

Following the routine NICU feeding protocol, all infants were preferably fed with their own mother’s milk (OMM) and, when this was not possible, with pasteurized human donor milk from the Milk Bank Unit. When the weight of the infants was ≥1500 g and OMM was unavailable, they received adapted preterm formula. Globally, the feeding patterns of the recruited infants were very heterogeneous, a fact that prevented the formation of well-defined feeding groups.

First spontaneously evacuated meconium after delivery was collected and, then, fecal samples were collected weekly during the first month of life and every 15 days until discharged from the NICU. However, only fecal samples from the first month of life were analyzed since more than half of the infants leave the NICU after one month of hospitalization (15 out of 26). As an exception (and when available), fecal samples beyond the first month were also analyzed for those infants that suffered a case of sepsis. OMM was extracted using electric pumps directly and stored either refrigerated for a maximum of 24 h or frozen for up to 6 months, while donor milk was pasteurized at 62.5 °C before storage. All the components of the pumps that were in direct contact with either the breast or the milk were washed with soap and water. Subsequently, the pumps were sterilized by boiling in water for 5 min or by using a microwave steam sterilizer bag following manufacter’s intructions.

All milks were incubated for 10 to 15 min at 37 °C to 40 °C and were administered to the infant through an orogastric or nasogastric feeding tube using a syringe (manual feeding) or a pump (automatic feeding). Milk samples were always collected after feeding from either the syringe or the extension set connected with the orogastric or nasogastric feeding tube at the same sampling times as the fecal ones. *S. marcescens* strains causing bacteremia were recovered from blood samples and identified at the Department of Microbiology of the Hospital Universitario 12 de Octubre. Immediately after collection, all the samples were stored at − 80 °C until analysis.

### Culture analysis of the samples

Suitable dilutions of food, meconium and fecal samples from the first month of life of the preterm infants were spread onto MacConkey agar media (BioMérieux, Marcy l’Etoile, France) for the isolation of *Enterobacteriaceae*. Plates were aerobically incubated at 37 °C for up to 48 h. Microbial counts were recorded as colony forming units (CFU) per gram of feces or as CFU per milliliter of milk, and transformed to log_10_ values before statistical analysis. At least one representative of each colony morphology type was grown in Brain Heart Infusion (BHI) broth (BioMérieux) and stored at − 80 °C in the presence of glycerol (30%, v/v) for further analysis.

### Bacterial identification

The *S. marcescens* isolates obtained from the fecal and feeding samples of the first month of life of the infants were identified by matrix-assisted laser desorption/ionization time-of-flight (MALDI-TOF) mass spectrometry in a Vitek-MS Instrument (BioMérieux).

### Genetic diversity among *S. marcescens* isolates

In order to avoid repeated isolates, the isolates were submitted to randomly amplified polymorphic DNA (RAPD) genotyping using the primer OPL5 (5′-ACGCAGGCAC-3′).

At least one representative of each different RAPD profile per infant was selected for PFGE assessment in a CHEF DR II apparatus (Bio-Rad, Birmingham, UK). The PFGE analysis of the *S. marcescens* isolates was carried out following the protocol described by Shi et al. [8]. In order to separate *Spe*I-digested fragments, the electrophoresis conditions were 5 to 25 s for 22 h. Dendograme analysis of PFGE patterns was performed using the UPGMA method based on Dice similarity and the INFO QUEST software (BioLine).

### Antimicrobial susceptibility testing

Antibiotic susceptibility for amoxicillin-clavulanate, piperacillin/tazobactam, cefuroxime, cefuroxime axetil, cefotaxime, ceftazidime, cefoxitin, cefepime, ertapenem, imipenem, amikacin, ampicillin, gentamicin, nalidixic acid, ciprofloxacin, tigecycline, and trimethoprim-sulphamethoxazole was determined in, at least, two representative isolates of each PFGE profile per infant. For this purpose, the Vitek 2 compact equipment and the Gram-negative antimicrobial susceptibility testing card ref. AST-N243 (BioMérieux) were used following the manufacturer’s recommendations. The breakpoints were selected following the Clinical and Laboratory Standards Institute guidelines [9].

### Statistical analysis

The statistical analysis was performed using R 2.15.3 (R-project, http://www.r-project.org). When data were not normally distributed, medians and interquartile ranges (Q1 and Q3) were calculated for all sampling times, and means and 95% confidence interval (95% CI) were used for normally distributed data. The Kruskal-Wallis test for non-normal data was used to evaluate the differences between sampling times. Fisher’s exact test was used to compare proportions. In all cases, *P* values of < 0.05 were considered to be significant.

## Results

The main demographic and clinical data of the 26 preterm infants included in the study are described in Table 1. The mean gestational age and birth weight were 27.7 weeks and 1167 g, respectively. All infants but two received ampicillin-gentamicin combination as a prophylactic treatment at birth. These and other antibiotics and antifungals (amphotericin B, amikacin, cloxacillin, erythromycin, fluconazole, fluorocytosine, meropenem, micafungin, teicoplanin, vancomycin) were occasionally used against a confirmed or suspected infection during the stay of the infants at the NICU. Seven infants suffered at least one sepsis episode during their hospital stay and *S. marcescens* was involved in two of them. Most of the infants required parenteral nutrition (*n* = 22), continuous positive airway pressure (CPAP) (*n* = 21), oxygenotherapy (*n* = 20) and mechanical ventilation (*n* = 16), whereas all of them were fed through enteral feeding systems. The median NICU and hospital stay was extended until 43 and 64 days, respectively.

**Table 1:** Means (95% Confidence Interval) of the Demographic Data and Clinical Characteristics of Preterm Infants Participating this Study

Characteristics	Mean (95% CI) or n (%)		
	Colonizated by *Serratia marcescens*	Not colonizated by *Serratia marcescens*	TOTAL
Infant	18 (69%)	8 (31%)	26 (100%)
Gestational age (wk)	27.8 (26.6;28.9)	27.4 (24.6;30.2)	27.7 (26.6;28.7)
Gender			
Male	6 (33%)	7 (87%)	13 (50%)
Female	12 (67%)	1 (13%)	13 (50%)
Birth weight (g)	1100.56 (927;1274.1)	1317.5 (804.5;1830.5)	1167 (987.3;1347.3)
Delivery mode			
Vaginal	6 (33%)	6 (75%)	12 (46%)
Cesarean section	12 (67%)	2 (25%)	14 (54%)
Antibiotherapy (days)			
No	1 (6%)	1 (13%)	2 (8%)
Yes	17 (94%)	7 (87%)	24 (92%)
<3 days	8 (44%)	3 (37%)	11 (42%)
>3 days	9 (50%)	4 (50%)	13 (50%)
Bronchopulmonary dysplasia			
No	11 (61%)	4 (50%)	15 (58%)
Yes	7 (39%)	4 (50%)	11 (42%)
Chorioamnionitis			
No	16 (89%)	8 (100%)	24 (92%)
Yes	2 (11%)	0 (0%)	2 (8%)
Sepsis	4 (22%)	3 (38%)	7 (27%)
Parenteral nutrition, *n*=22 (days)	7 (5-9.5)^a^	11 (5.5-12.8)^a^	7.5 (5-12.8)^a^
Enteral feeding tube (days)	58 (45.7;69.9)	61.5 (21.9;101.1)	59 (46;72)
Mechanical ventilation, *n*=16 (days)	2 (0.8-9.5)^a^	37 (35-37)^a^	8.5 (1-35.5)^a^
CPAP, n=21 (days)	11 (5.3-45.8)^a^	5 (1.8-22)^a^	9 (5-45)^a^
Oxygenotherapy, n=20 (days)	27 (2.5-76.3)^a^	41 (2-92)^a^	32 (2-84)^a^
NICUs (days)	43 (27.3-66)^a^	30.5 (7.8-85.8)^a^	42 (18.5-77.8)^a^
Hospital stay (days)	68 (44.8-81.3)^a^	39 (27-105.5)^a^	64 (41-90)^a^

^a^ Median (IQR)

Most of the infants (69%) presented *S. marcescens* in, at least, one of their fecal samples, and 54% of them harbored this species in 2 or more samples. Some differences were observed in the demographic and clinical characteristics of the infants depending on their colonization or not with *S. marcescens*. Colonization level was statistically higher in females (*P* = 0.012), infants born by C section (*P* = 0.049) and those that required less days of mechanical ventilation (*P* = 0.023). In addition, and although they did not reach statistically significant values, three trends were observed among infants colonized with this species; (a) they remained for a longer time at the NICU (43 versus 30.5 days) and hospital (68 versus 43 days); (b) their requirement of antibiotherapy and CPAP was higher; and (c) clinical variables associated with prematurity, such as birth weight and chorioamnionitis were also associated with *S. marcescens* presence (Table 1).

*S. marcescens* could not be isolated from any of the meconium samples. However, it was isolated (and at a high concentration) from feces of 5 infants (28%) only one week after birth (Table 2). The number of *S. marcescens*-colonized infants as well as the concentration of this species in the positive samples increased in the samples collected two and three weeks after birth (Table 2). Later, both parameters decreased in the samples collected in the fourth week after birth (Table 2).

**Table 2:** *Serratia marcescens* and other *Enterobacteriaceae* Isolated from Meconium and Fecal Samples from the first month of life of the infants

		Meconium (*n*=16)	1st week feces (*n*=18)	2nd week feces (*n*=23)	3rd week feces (*n*=24)	4th week feces (*n*=11)	*P* value
*S. marcescens*	n (%)	0 (0%)	5 (28%)	11 (48%)	13 (54%)	6 (55%)	<0,05**
	Microbial Counts Median (IQR)	-	8,462 (8,332-9,230)	9,079 (8,942-9,593)	9,333 (8,683-9,557)	8,676 (7,874-9,195)	0,114*
Other *Enterobacteriaceae*	n (%)	1 (6%)	14 (78%)	19 (83%)	20 (83%)	11 (100%)	<0,05**
	Microbial Counts Median (IQR)	7,267 (7,267-7,267)	9,154 (8,484-9,515)	9,322 (9,007-9,487)	9,145 (9,038-9,517)	9,362 (8,971-9,660)	0,415*

**Fisher test

*KW test

**Fig. 1:**
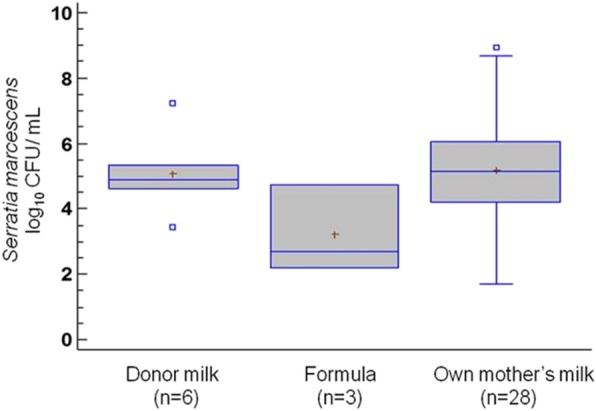
Mean (IC 95%) concentration of *S. marcescens* (log_10_ CFU/g) in the different milk types after their pass through the feeding tubes. Donor milk: 5.06 (3.75; 6.36); formula: 3.20 (− 0.13; 6.53); own mother’s milk: 5.18 (4.51; 5.84)

Culture analysis of the feeding samples obtained after their pass through the enteral feeding tubes revealed that 33, 17 and 20% of OMM, donor milk and formula milk samples, respectively, contained *S. marcescens*. Mean microbial counts oscillated from 3.20 CFU/ml in formula samples to 5.18 CFU/ml in OMM ones, however no statistical differences were found between feeding types (Fig. 1).

**Fig. 2:**
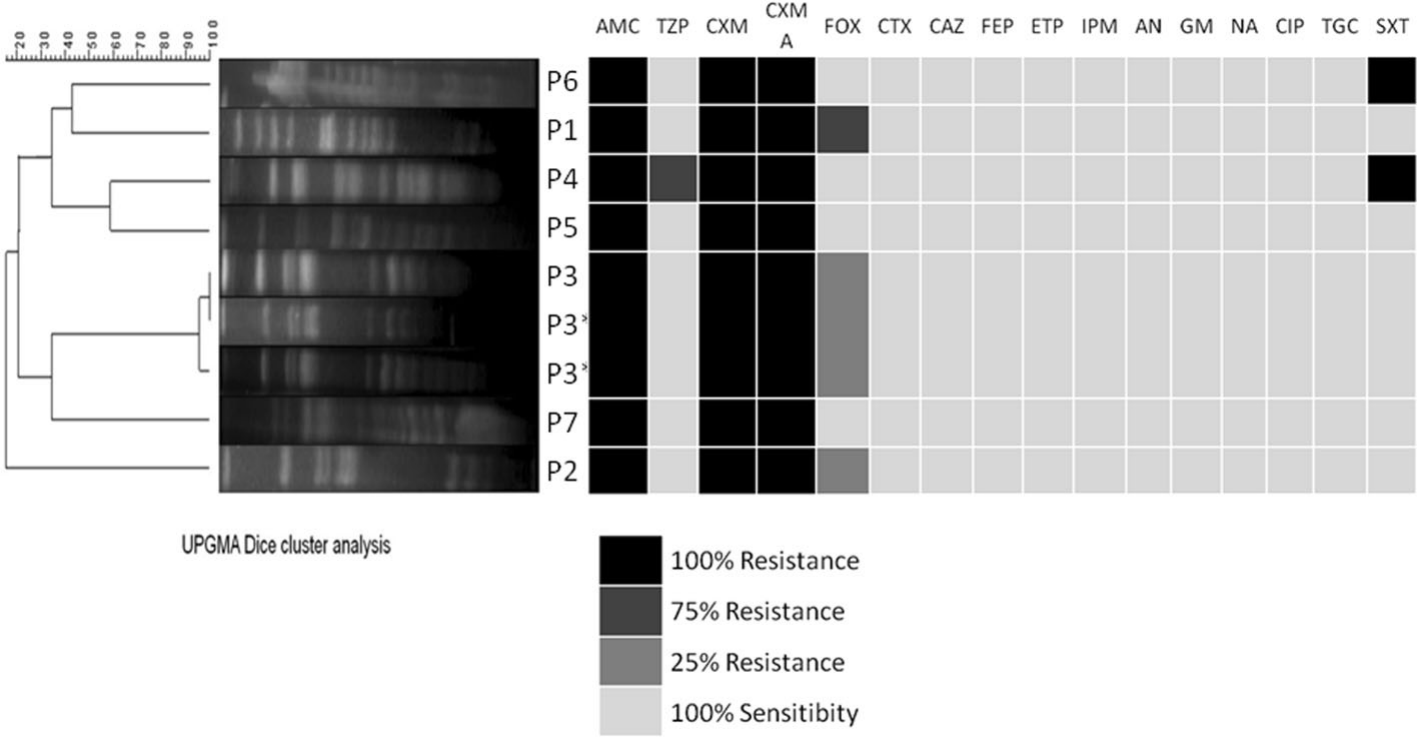
Cluster analysis and antibiotic susceptibility of the different *S. marcescens* PFGE profiles obtained in this study. P1-P7: Profiles 1 to 7; P3*: Profile 3 obtained from the blood of the infants suffering bacteriemia. The cluster analysis was performed using the UPGMA method based on Dice similarity coefficient. Results of the antibiotic susceptibility assays are expressed by percentage of resistance using a color scale. AMC, amoxicilin/clavulanic acid; TZP, piperacillin/tazobactam; CXM, cefuroxime; CXM A, cefuroxime; FOX, cefoxitin; CTX, cefotaxime; CAZ, ceftazidime; FEP, cefepime; ETP, ertapenem; IPM, imipenem; AN, amikacin; GM, gentamicin; NA, nalidixic acid; CIP, ciprofloxacin; TGC, tigecycline; SXT, trimethoprim/sulfamethoxazole

**Fig. 3:**
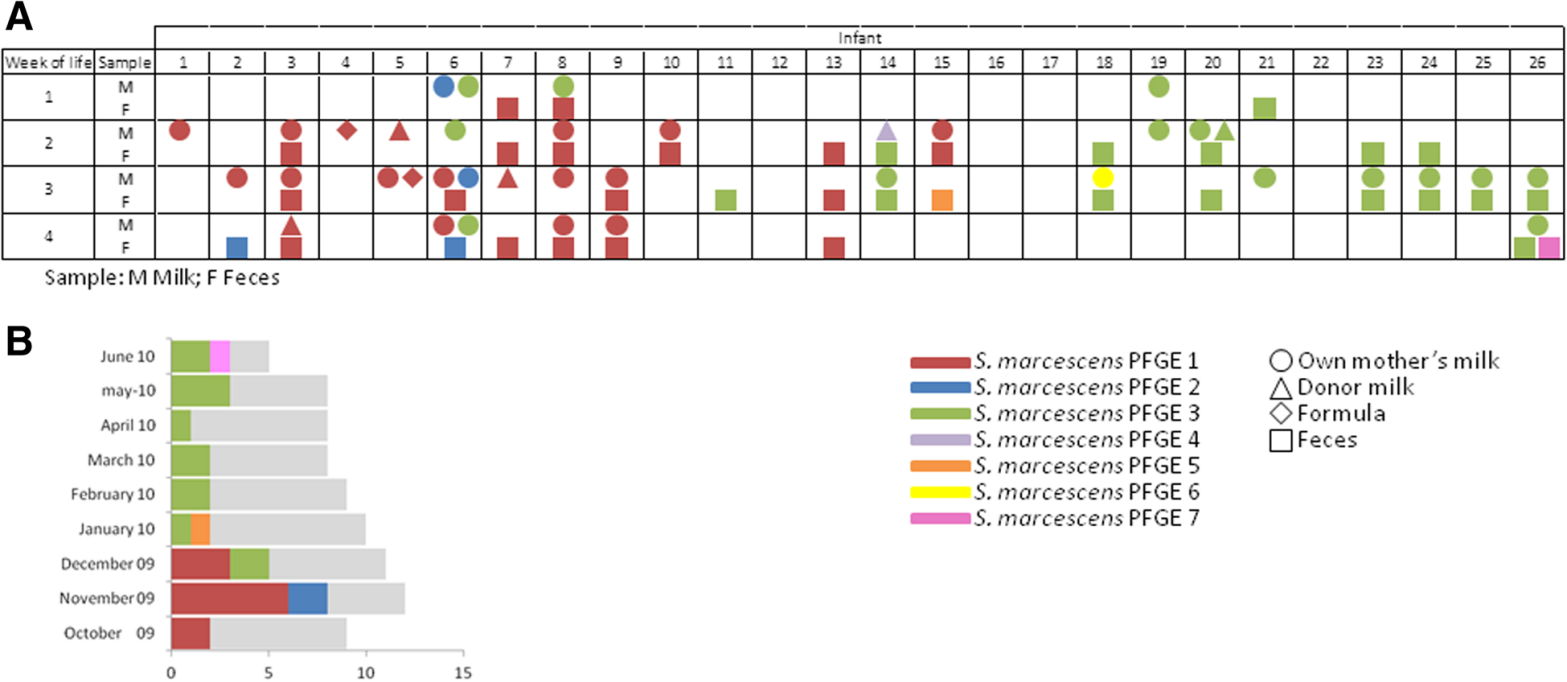
(**a**) PFGE profiles of *S. marcescens* in feces and milk samples of the infants along the first four weeks of life. (**b**) Colonization rates of each PFGE profile of *S. marcescens* along the study period

A total of 179 isolates of *S. marcescens* were isolated from the fecal and feeding samples collected during the first month of life of the infants. RAPD analysis allowed a first reduction of the initial collection to 123 isolates, which were subsequently submitted to PFGE profiling. Cluster analysis of the PFGE profiles allowed the classification of all the isolates into 7 different *S. marcescens* strains (profiles 1 to 7) (Fig. 2). PFGE patterns 1, 2 and 3 clustered ~ 95% of the isolates obtained from all the samples throughout the study period. In some of the infants the *S. marcescens* strains appeared first in feeding samples and, subsequently, in feces, but in approximately half of them this relationship could not be determined. Colonization rate by *S. marcescens* oscillated during the study period showing a pronounced peak in November and December 2009 (67 and 45% of the infants, respectively). Profile 1 was the dominant during the first 3 months, both in fecal and feeding samples, while profiles 2 and 3 were isolated from the feeding samples one and two months, respectively, before they could be detected in fecal samples. In the first months of the study, profile 1 reached colonization rates of 100, 75 and 67% of the infants that presented *S. marcescens* in their feces. However, after the fifth month of the study period, a significant shift in the *S. marcescens* colonization pattern was observed and PFGE profile 3 became the dominant or unique strain that could be isolated during the following 5 months. PFGE profiles 4 to 7 were only occasionally isolated from the biological samples analyzed in this study. Profiles 5 and 7 were exclusively detected from fecal samples, while profiles 4 and 6 were exclusively isolated from feeding samples (Fig. 3).

As stated above, two infants suffered a *S. marcescens* sepsis and one of them suffered two distinct episodes. Both infants were born with a gestational age (26 and 24 weeks, respectively) and birth weight (920 and 750 g, respectively) lower than the mean of the studied population (27.7 days and 1167 g, respectively). In one of these infants the bacterial counts in the fecal samples collected in the weeks preceding the sepsis episode was more than one logarithmic unit higher than the media obtained in this cohort. One of the PFGE profiles (profile 3) included those isolates that caused bacteremia in the two infants (Fig. 2). Globally, PFGE pattern 3 included 45% of the *S. marcescens* strains isolated in the study. Profile 3 could be isolated from both blood and fecal samples of the infected infants and presented the same antibiotic susceptibility profile. When they suffered the sepsis episodes they were fed with a mixture of OMM and donor milk. Feeding samples were also contaminated with the strain but, in both cases, the strain appeared first in the fecal sample and, later, in the feeding samples.

The antibiotic susceptibility profile of all the strains included resistance to amoxicillin/clavulanic acid, cefuroxime and cefuroxime axetil. In addition, some of the strains showed variable susceptibility to cefoxitin (profiles 1, 2 and 3), trimethoprim/sulfamethoxazole (profiles 4 and 6) and piperacillin/tazobactam (profile 4) (Fig. 3).

## Discussion

The presence of *S. marcescens* in the hospital environment as well as the number of infections caused by these bacteria in immunocompromised patients (including urinary tract infections, pneumonia, skin and soft tissue infections, septicemia, brain abscesses and meningitis) have increased during the last decades over the world [10–13]. *S. marcescens* has become an important cause of nosocomial infections especially in NICUs [10, 14, 15], where it can lead to mortality rates as high as 44% [11, 16, 17]. However, epidemiological data about the source of most of the *S. marcescens* outbreaks remained unknown [14, 18]. This fact can be explained on the basis of the ubiquity and resistance of this species; it can survive on inanimate surfaces (including medical devices and breast pumps) and even in antiseptic solutions for long periods of time [19–24]. In addition, they colonize the respiratory and/or gastrointestinal tract of symptomatic and asymptomatic hosts [12, 17, 25]. The current study was directed to elucidate the colonization rate in preterm neonates, one of the populations more affected by *S. marcescens* infections.

Our results showed that *S. marcescens* colonizes the preterm infant gut within the first days after birth, following a colonization pattern very similar to that observed in other members of the Family *Enterobacteriaceae* [6, 26]. Globally, 69% of the infants were colonized by *S. marcescens* at least in a sampling time during the studied period. The colonization rate increased to ~ 75% when only infants born at < 30 gestational weeks and < 1500 g were considered. A higher colonization, together with a more immature immune system and an altered intestinal barrier, may explain a higher predisposition of very preterm neonates to *S. marcescens* sepsis [10, 11, 14, 15, 18].

As stated above, feeding samples were obtained after their pass through the external part of the feeding tubes. Feeding tubes were not routinely replaced during the study and different feed types (OMM, donor milk, infant formula) could pass through the same tube. Donor milk and formula, in contrast to OMM, were sterile before its administration, although were contaminated with *S. marcescens* after their pass through the feeding tubes. OMM was expressed using electric pumps and stored refrigerated until the next feeding time. Human milk is a source of commensal and probiotic bacteria [27] but milk pumps can become heavily contaminated during manipulation and rinsing with water. Different bacterial species, such as *Acinetobacter baumannii*, *Escherichia coli*, *Klebsiella pneumonie, Pseudomonas aeruginosa, Staphylococcus aureus* or *S. marcescens*, have been associated with NICUs outbreaks due to contaminated pump-expressed milk [20, 21, 24]. In this study, the high contamination found in donor and formula milk revealed the contributing role of the external feeding tube; the gastric content of preterm neonates is routinely aspirated before each feeding in order to know the residual gastric volume and, subsequently, it is re-inoculated back to the stomach; we hypothesize that such a practice may spread bacteria that rapidly colonize the gastrointestinal gut of preterms, such as *S. marcescens*, to the feeding tubes. Since such tubes are rich in nutrients and are located at a temperature that is optimal for growth of enterobacteria, this can lead to a rapid colonization of the whole feeding system. A limitation of the study is that the feeding samples could not be analyzed before their pass through the feeding tubes; therefore, environmental contamination, including contamination during OMM collection and/or during delivery of either OMM, donor milk or infant formula cannot be ruled out as a source of *S. marcescens* in this study. Independently of the original source, feeding tubes are excellent sites for bacterial biofilm formation, preferentially by members of the Family *Enterobacteriaceae,* and could act as a reservoir of these bacteria in the NICU [26, 28–30]. The nasogastric tubes replacement protocol in the hospital was changed after observing the contamination level of the feeding samples [26].

In the two infants that suffered sepsis by *S. marcescens*, the respective strains appeared first in the fecal sample and, later, in the feeding samples. This suggests that OMM was not the original source of the strains and that, after initial gut colonization and subsequent colonization of the feeding tubes by *S. marcescens,* OMM samples were contaminated after their transit through the feeding tubes.

All strains tested in our study were susceptible to tigecycline, carbapenems, aminoglycosides and quinolones. In addition, most of cephalosporins and beta-lactams included in the antibiogram remained active against this pathogen. Although similar results have been obtained previously [16], other studies have shown a wider spectrum of antibiotic resistances, particularly against carbapenems and cephalosporins, among strains causing outbreaks [15, 18].

The ubiquity of *S. marcescens*, its biofilm-forming capacity, its close and opportunistic association with prematurity, and the increasing cases and outbreaks frequency at NICUs, should encourage biomedical research focused on its epidemiology (including the availability of MLST or similar schemes and the identification of risk clones), genome sequencing, predisposing factors (host, microbial, ecological and medical factors), and measures to avoid that this species may become a major cause of preterm infections and sepsis.

The current study was considered in order to provide valuable information about *Serratia* population dynamics in a NICU. Considering that, despite it is a well-known cause of outbreaks in NICUs, there is still limited information about its reservoirs and specific control measurements. Our results suggested enteral feeding tubes act as important reservoirs to keep the *S. marcescens* population in the NICU. Therefore, enteral feeding tubes colonization by potential pathogens requires a stronger attention. The search of new materials or nanostructures that limited the bacterial attachment in medical devices and other new therapies and preventive approaches to reduce nosocomial infection should be encouraged. Understanding the epidemiology and clonality of *S. marcescens* has implications to control its spread on the NICU environment and prevent future infections in this sensitive population.

## Conclusions

*S. marcescens* is a bacterial species closely associated to the NICU environment and recently has emerged as a causing agent of sepsis in preterm infants. It can be frequently isolated from preterm’s feces, however only some genetic lineages seem to be associated with sepsis. Between the reservoirs of the microorganism in the NICU enteral feeding tubes are remarked here as important focus to infant’s gut colonization, sothat its hygiene and replacement must be maximize.

## Data Availability

The datasets generated during the current study are available from the corresponding author on reasonable request.

## Abbreviations

CFU: Colony Forming Units
CPAP: Continuous Positive Airway Pressure
ELBW: Extremely Low Birth Weight
MALDI-TOF: Matrix-Assisted Laser Desorption/Ionization Time-Of-Flight
MLST: Multi Locus Sequence Type
NEC: Necrotizing Enterocolitis
NICU: Neonatal Intensive Care Unit
OMM: Own Mother’s Milk
PFGE: Pulse Field Gel Electrophoresis
RAPD: Randomly Amplified Polymorphic DNA
VLBW: Very Low Birth Weight
